# Dietary Minerals and Incident Cardiovascular Outcomes among Never-Smokers in a Danish Case–Cohort Study

**DOI:** 10.3390/ijerph21070932

**Published:** 2024-07-17

**Authors:** Victoria Fruh, Tesleem Babalola, Clara Sears, Gregory A. Wellenius, Thomas F. Webster, Koren K. Mann, James Harrington, Anne Tjønneland, Ole Raaschou-Nielsen, Birgit Claus Henn, Jaymie R. Meliker

**Affiliations:** 1 Department of Environmental Health, Boston University School of Public Health, 715 Albany Street, Boston, MA 02118, USAtwebster@bu.edu (T.F.W.);; 2 Program in Public Health, Department of Family, Population, & Preventive Medicine, Stony Brook University, Stony Brook, NY 11794, USA; tesleem.babalola@stonybrookmedicine.edu (T.B.);; 3 Division of Environmental Medicine, Department of Medicine, University of Louisville, Louisville, KY 40292, USA; clara.sears@louisville.edu; 4 Department of Pharmacology and Therapeutics, McGill University, Montreal, QC H3A 0G4, Canada; 5 Center for Analytical Science, Research Triangle Institute, Research Triangle Park, NC 27709, USA; 6 Danish Cancer Society Research Center, 2100 Copenhagen, Denmark; annet@cancer.dk (A.T.);; 7 Department of Public Health, University of Copenhagen, 1353 Copenhagen, Denmark; 8 Department of Environmental Science, Aarhus University, Frederiksborgvej 399, 4000 Roskilde, Denmark

**Keywords:** elements, nutrients, minerals, dietary intake, cardiovascular disease, cardiovascular epidemiology

## Abstract

**Background:** Diet is known to impact cardiovascular disease (CVD) risk, but evidence for the essential minerals of magnesium (Mg), calcium (Ca), and potassium (K) is inconsistent. **Methods:** We conducted a case–cohort study within a non-smoking subgroup of the Danish Diet, Cancer and Health cohort, a prospective study of 50–64-year-olds recruited between 1993–1997. We identified incident heart failure (HF), acute myocardial infarction (AMI) and stroke cases through 2015 with an 1135-member subcohort. We measured the dietary intake of minerals, also known as elements, and calculated a combined dietary intake (CDI) score based on joint Ca, Mg and K intakes (mg/d) from Food Frequency Questionnaires. We estimated adjusted hazard ratios (HRs) with Cox proportional hazard models. **Results:** Most HRs examining associations between CDI score and CVD were null. However, the third quartile of CDI was associated with a lower risk for heart failure (HR: 0.89; 95% CI: 0.67, 1.17), AMI (HR: 0.79; 95% CI: 0.60, 1.04), and stroke (HR: 0.63; 95% CI: 0.44, 0.88). **Conclusions:** We did not find consistent evidence to suggest that higher levels of essential minerals are associated with incident HF, AMI, and stroke, though results suggest a potential U-shaped relationship between select minerals and CVD outcomes.

## 1. Introduction

Cardiovascular disease (CVD) is the leading cause of death worldwide, contributing to more than 17 million deaths per year [1]. Acute myocardial infarction (AMI), stroke, and heart failure (HF) are prominent CVD conditions. AMI occurs when there is an abrupt blockage of blood flow to the heart muscle [2,3], and the overall incidence of AMI is approximately 800,000 cases per year in the United States (U.S.), with approximately 695,547 deaths per year [4]. Globally, the prevalence of AMI is approximately 3 million cases [4]. An estimated 795,000 stroke cases are reported each year in the U.S., resulting in 146,000 deaths, and the Global Burden of Diseases (GBD) estimates reported stroke as the second leading cause of death and disability, with 6.6 million deaths [5]. For HF, the global prevalence is 64.34 million cases (8.52 per 1000 inhabitants), resulting in 9.91 million years of healthy life lost due to disability and an estimated 346.17 billion dollars in economic burden [6,7]. 

While dietary intake and physical activity are known modifiable risk factors for CVD related conditions, a growing literature points to the potential role of environmental elements (commonly referred to as minerals) in association with CVD [3,8,9,10,11,12]. Mechanistically, the essential minerals of magnesium (Mg), calcium (Ca), and potassium (K) may play important roles in processes that slow CVD progression. Mg is involved in crucial metabolic processes for human health including synthesis of DNA and RNA, cell signaling, serving as a co-factor for enzymes, and regulating blood pressure [13,14]. Mg is also important in its regulation of smooth vascular muscle tone, muscle contraction, heart rhythm and endothelial function [14,15]. K may assist in the reduction of blood pressure and facilitate vasodilation [16]. Ca is essential in myocardium contraction, serves as an enzyme co-factor, and may influence plaque stabilization [17,18,19]. On the other hand, Ca deposition in the blood vessels can adversely impact cardiovascular health outcomes [17,18,19], and the optimal level of dietary Ca for heart health remains unknown [17,18]. While the aforementioned research [17,18,19] has demonstrated beneficial relationships between these elements and mechanisms involved in cardiovascular health [8,9,11,12], literature for Ca [17,19,20] and Mg [14,21,22,23] in relation to specific CVD outcomes is still inconclusive. Additionally, research on the associations between AMI risk and the essential minerals Ca, Mg and K remains sparse, with inconsistent findings [24,25,26,27,28]. Furthermore, no known studies to date have examined AMI, HF and stroke in association with a more comprehensive combined dietary intake score for mineral intake while also comparing these findings to urinary measures.

Therefore, in this study, we aimed to evaluate the association between dietary intake of Ca, Mg, and K and the incidence of HF, AMI, and stroke. We hypothesized protective associations between essential minerals and CVD outcomes. We built on previous work by evaluating associations between dietary intake of the essential elements (henceforth referred to as minerals) of Ca, Mg, and K with incidence of HF, AMI and stroke among a subgroup of never smokers from Denmark. We investigated the combined dietary intake score [29,30] as a more comprehensive metric of mineral intake in addition to individual mineral intake. Never smokers were selected to eliminate possible confounding from cigarette smoking and related behaviors, as smoking has been associated with blood and urinary mineral levels and CVD independently [31,32,33]. As a secondary analysis, we investigated the association between essential minerals measured in urinary concentrations and CVD outcomes and compared results from dietary intake versus urinary mineral measures. 

## 2. Materials and Methods

### 2.1. Study Participants

We evaluated data from participants enrolled in the Danish Diet, Cancer, and Health (DCH) study. Details of enrollment and recruitment for this cohort have been previously published [34]. The current study builds on prior analyses from the DCH case-cohort population that evaluated associations between cadmium (Cd) and incident HF, AMI, and stroke [35,36,37]. Briefly, the DCH cohort is a prospective study consisting of 57,053 50–64-year-old participants recruited between 1993 and 1997 from two areas of Denmark (Aarhus and Copenhagen). Participants were required to have no cancer diagnosis at baseline. Participants completed questionnaires about lifestyle factors and food frequency, completed a physical examination, and provided biological samples at baseline, including urinary samples. Participants were followed over time through Denmark health registers [38]. For the present analysis, inclusion criteria consisted of participants who self-reported never smoking at baseline. We excluded participants missing urine samples or smoking status information, as previously described [37]. Complete case analysis was conducted in our study as little data were missing on minerals (<3%) nor covariates (ranging from 0% for age to <2% for physical activity). Of the 57,053 DCH study participants, 19,394 were never smokers and met the inclusion criteria for this analysis [37]. The DCH study has been conducted in accordance with the Helsinki Declaration and approved by the research ethics committee for Copenhagen and Frederiksberg. Participants provided written informed consent. This analysis was based on de-identified data and determined to be non-human subject research by the Institutional Review Boards (IRBs) at Boston University and Stony Brook University.

### 2.2. Acute Myocardial Infarction (AMI) Cases 

We identified all cases of AMI among all eligible participants who never smoked during follow-up through 2015. We identified AMI cases by using individuals’ unique civil registration numbers and the Danish National Patient Registry (NPR) [38], as described previously [37]. We identified participants registered with a primary or secondary diagnosis of AMI in the NPR using ICD codes: ICD-8 410–410.99, 427.27 and ICD-10: I21.0-I21.9 and I46.0-I46.9 [38]. Following the case-cohort design, we subsequently selected a random sample of never-smokers, consisting of 600 men and 600 women (total n = 1200), to obtain a representative subcohort used for all outcomes. We conducted a complete case analysis among 1135 members of the randomly selected subcohort and 776 AMI cases (enrollment through 2015). There were 56 participants within the subcohort that were also AMI cases, resulting in an analytic cohort of 1855 (1135 + 776 − 56 = 1855). The median follow-up within the AMI case–cohort was 18.8 years. 

### 2.3. Stroke Cases

Among eligible DCH participants, we identified all stroke cases and diagnosis dates in the Danish National Patient Registry (NPR) through individual personal identification numbers using the ICD codes: ICD10: I60, I61, I63, I64. Using a complete case–cohort analysis approach as described above for AMI cases, a total of 502 incident cases of stroke were identified within the follow-up period (enrollment through 2009). The subcohort, as described above, included 1135 participants. There were 43 participants within the subcohort that were also stroke cases, resulting in an analytic cohort of 1594 (1135 + 502 − 43 = 1594). The median follow-up time for stroke was 13.6 years. 

### 2.4. Heart Failure (HF) Cases

We identified all incident HF cases among eligible DCH participants and diagnosis dates in the Danish National Patient Registry (NPR) through individual personal identification numbers using the ICD codes: ICD-10 codes I50.0-I50.9 and I11.0. Following the same case-cohort approach described above, 893 HF cases were identified (enrollment through 2015). The subcohort, as described above, included 1135 participants. There were 59 participants within the subcohort who were also HF cases, resulting in an analytic cohort of 1969 (1135 + 893 − 59 = 1969). The median follow-up time for HF was 19.8 years. 

### 2.5. Food Frequency Questionnaire (FFQ)

The semi-quantitative food frequency questionnaire (FFQ) consisted of a validated food frequency and consumption questionnaire with 192 items. The frequency of food consumption responses ranged from “never” to “eight times or more per day.” FoodCalc [39] was used to estimate the average food and mineral intake for each participant, including Ca, Mg and K intake levels (mg/day), with a method that accounts for gender-specific portion sizes and frequency of consumption [34]. Additionally, data on the use of dietary supplements were collected in the form of open-ended questions about supplement brand and intake dose, as well as categorical questions on intake frequency (i.e., number of months over the last year that supplements were taken and supplement intake within the past month) [34]. Mineral content information for dietary supplements was obtained from producers/distributors by brand and product [34]. 

### 2.6. Combined Dietary Intake Score

Given that Ca, Mg and K minerals exist together in various foods, we aimed to understand associations between combined dietary mineral intake and CVD outcomes. We, therefore, calculated a combined dietary intake (CDI) score based on joint Ca, Mg and K intakes (mg/d) from the FFQ. The CDI score was calculated by assigning points based on the quartile of the distribution for each mineral and summing those points across minerals for each participant based on prior evidence of associations between this score and stroke incidence [29]. As an example, if a participant’s dietary mineral intake was in the lowest quartile for all three minerals (Ca, Mg and K), the participant received a score of 1 point for each mineral, and the CDI summed to 3 total points (minimum CDI score). Participants with levels in the highest quartile for each mineral (Ca, Mg and K) received a score of 4 points for each mineral, for a total of 12 points (maximum CDI score). Accordingly, the CDI score ranged from 3–12 points.

### 2.7. Urinary Minerals, Creatinine, and Cotinine 

Participants provided spot urine samples at enrollment. Samples were collected in a polypropylene cup using a technique free from trace metals and stored at −150 °C in a samples biobank [34]. Samples were anonymized and subsequently sent to RTI International’s Trace Metals Laboratory (Research Triangle Park, NC) for analysis of urinary minerals, as previously described [37]. Details about sample preparation, analysis of trace Cd, and quality control have been previously published [35]. To analyze the bulk minerals Ca, Mg and K in urine samples, urine was diluted by a factor of 100 with a 2% nitric acid solution and analyzed using an iCAP 7600 ICP-OES system (Thermo Scientific, Waltham, MA, USA) equipped with a Peltier-cooled glass cyclonic spray chamber and 2 mm quartz injector. The National Institute of Standards and Technology (NIST, Gaithersburg, MD) traceable stock standards were used for calibration. RTI International monitored multiple wavelengths to verify the absence of analytical interferences. Analytical accuracy was monitored by preparing and analyzing replicates of Seronorm Trace Minerals in Urine-certified Reference Material Level 1 (Lot 1706877; recovery ranging from 90.2–100%; precision ranging from 0.5–0.6% RSD). Incurred sample reanalysis, performed for ~5% of study samples (n = 149 samples), demonstrated agreement with correlation coefficients of *r* = 0.91–0.96 after the exclusion of obvious outliers. We measured creatinine colorimetrically and used the Jaffe reaction with a Cayman Chemicals (Ann Arbor, MI) Creatinine Assay Kit, as described previously [37], to account for urinary dilution. To calculate creatinine-standardized concentrations of minerals in urine, we divided urinary mineral concentrations by grams of creatinine. We also measured urinary cotinine as an indicator of tobacco smoke exposure. Cotinine was measured with a cotinine ELISA bioassay kit developed by Abnova Corporation (Taipei, Taiwan). 

### 2.8. Covariates 

Participants completed questionnaires about lifestyle factors (i.e., smoking status, physical activity, alcohol use) and socioeconomic characteristics (i.e., education status) at enrollment, as published previously [34]. Passive environmental tobacco smoke exposure was defined in this study as low passive smoke exposure (i.e., no smoker in the home from 20 years of age or older, and passive environmental tobacco smoke exposure at work <4 h/day from 20 years of age or older) vs. high passive smoke exposure (exposure otherwise) [40,41,42]. Women reported menopause status and parity. We recorded anthropometry and blood pressure based on physical examination at baseline. 

We additionally calculated a lifestyle factor index (LFI), adapted from previous work in the DCH cohort and described previously [43]. The LFI was generated based on previous international public health recommendations [44,45,46]. The LFI allowed us to include data from multiple lifestyle covariates in our final models without collinearity concerns, given that lifestyle factor covariates were highly correlated as individual factors, including higher correlations between dietary factors as previously published [43]. Four lifestyle factors were included in the LFI for never-smokers, and one point for each of the following recommendations was assigned, with a maximum of four points: (1) at least 30 min of physical activity per day; (2) alcohol intake of less than or equal to 7 drinks/week for women, or less than or equal to 14 drinks/week [47] for men; (3) waist circumference of less than 88 cm for women, or less than 102 cm for men; and (4) all of the following dietary recommendations adhered to: consuming greater than or equal to 600 g of fruit and vegetables per day, less than or equal to 500 g of red or processed meat per week [47] and greater than or equal to 3 g of dietary fiber per MJ (megajoule) of dietary energy [43]. Based on the summation of these scores, the LFI ranged from zero (least healthy) to four (most healthy) points per person.

### 2.9. Statistical Analysis

Descriptive statistics were computed for the analytic cohort. We calculated Spearman correlation coefficients between mineral levels from dietary intake (FFQ) and urinary analysis. In the main analysis, we evaluated associations between CDI score and HF, AMI and stroke as individual endpoints [48]. We used the Prentice case–cohort approach with Cox proportional hazards models [49]. In these models, participants contributed person-time from their DCH enrollment date through the date of CVD diagnosis or the date of the censoring event (emigration, death, or end of follow-up), whichever was earliest. We included age as the timescale in these models [37,50]. Similar to previous DCH analyses and consistent with case–cohort methods [37,48,51], cases among DCH participants who were not included in the subcohort were not considered to be at risk for the combined outcome until immediately preceding the date of diagnosis. Mineral intakes were categorized into quartiles to maximize power for comparisons and because there are no established cut-point values for “low”, “normal”, or “high” intake values. Analyses were conducted in R version 3.6.2 with the survival package to account for the case–cohort study design.

We estimated adjusted hazard ratios (HRs) and 95% confidence intervals (95% CIs) within Cox proportional hazard case-cohort models, adjusted for potential confounders selected based on *a priori* knowledge of the relationships between covariates and minerals, and between covariates and CVD outcomes. Covariates included in our fully adjusted models were not highly correlated. Fully adjusted models included biological sex (male vs. female), years of education [categorical variable: low (≤7 years), medium (8–10 years), and high (>10 years)], passive smoking (high passive smoke exposure vs. no/low passive smoke exposure), and lifestyle factor index (categorical variable: 0–4, where 0 indicates less healthy lifestyle factors and 4 indicates more healthy lifestyle factors) as covariates. 

We modeled the CDI score as a categorical variable (i.e., quartiles) to allow for non-linearity in associations with CVD outcomes. We also modeled associations between individual mineral intakes and CVD outcomes, where separate models were fit for each mineral given that pairwise correlations were moderate (0.63–0.71) to high (0.91–0.93) for some minerals (Table S1).

In secondary analyses, we evaluated the associations between urinary mineral concentrations and CVD outcomes using creatinine-adjusted urinary levels of Ca, Mg and K. We modeled urinary mineral concentrations as categorical variables (quartiles) to allow for non-linearity. Fully adjusted models (as described above) additionally included energy intake (continuous, kilojoules/day or kJ/d). It was necessary to adjust for energy intake in urinary models only rather than in dietary intake models, given that dietary intake is a proxy of energy intake and that these variables are highly correlated. For both main and secondary models, we additionally stratified by sex to assess potential differences in associations by biological sex.

### 2.10. Sensitivity Analyses

We conducted a sensitivity analysis to account for the total intake of minerals from both regular dietary intake, as captured by the FFQ, and from dietary supplements. We created a combined total intake (CTI) score by first summing across dietary intake (mg/d) and supplement intake (mg/d) for each individual mineral, then categorizing each mineral into quartiles, and finally summing quartiles across minerals to create a summed total intake, using the same process as above for CDI score. We estimated associations between CTI and CVD outcomes in the same way as described above.

We completed several additional sensitivity analyses to investigate the robustness of our findings for primary analyses of dietary intake: (1) we restricted to participants with no self-reported hypertension, hypercholesterolemia, or diabetes at baseline, as these outcomes may be on the causal pathway to CVD; (2) we restricted to participants with cotinine levels under the 90th percentile to determine if findings were similar among participants with indicators of lower tobacco smoke exposure, in addition to all participants self-identifying as never-smokers; (3) we restricted to women who were post-menopausal to assess potential evidence of differences in the association among women by menopause status; (4) we compared minimally adjusted models without LFI as a covariate to models with LFI and to models with LFI components included as independent variables, to investigate the extent of confounding by LFI; (5) we additionally adjusted for multivitamin supplement use (yes vs. no) and whole grain cereal intake (grams); (6) we compared creatinine-standardized vs. creatinine-adjusted results; and (7) in addition to conducting analysis for CVD outcomes individually, we evaluated the composite outcome of AMI or stroke (AMI-Stroke), given the atherosclerotic mechanism of action common to both of these conditions [52,53]. The definition of the AMI-Stroke outcome followed the ICD-10 coding listed above for AMI and stroke. 

## 3. Results 

The mean age at the time of enrollment for the subcohort and cardiovascular cases ranged from 56 to 58 years, and over half of the participants were men (50–60% across the different case populations). The mean BMI was 26.4 for the subcohort group, 26.8 for HF cases, and 27.4 among AMI and stroke cases (Table 1). Compared with the subcohort, participants diagnosed with CVD outcomes tended to have lower lifestyle factor index (LFI) scores, were less likely to be married or employed, and had fewer years in school (Table 1). Among those who self-identified as female, a higher proportion of CVD cases were post-menopausal (90.0–92.1%) when compared to the subcohort (81.9%) (Table 1). HF cases had similar median CDI scores and slightly higher median Ca, Mg, and K intake values compared to stroke or AMI cases or the subcohort (Table 2).

Dietary Mg and dietary K were highly correlated (Spearman *r* coefficient range of 0.91–0.93, by CVD outcome). Dietary Ca was moderately correlated with dietary K and Mg (*r* coefficient range 0.68–0.71). Spearman correlations between dietary and creatinine-adjusted urinary measures of the *same* mineral were generally weak (range of *r* coefficients: −0.002 for Mg to 0.07 for K and Ca) (Table S1). Urinary mineral levels were moderately correlated with other urinary minerals (*r* coefficient range of 0.57–0.63 for urinary Mg–Ca, 0.33–0.45 for urinary Mg–K, and 0.29–0.32 for urinary Ca–K) (Table S1).

### 3.1. Main Analysis

**Figure 1:**
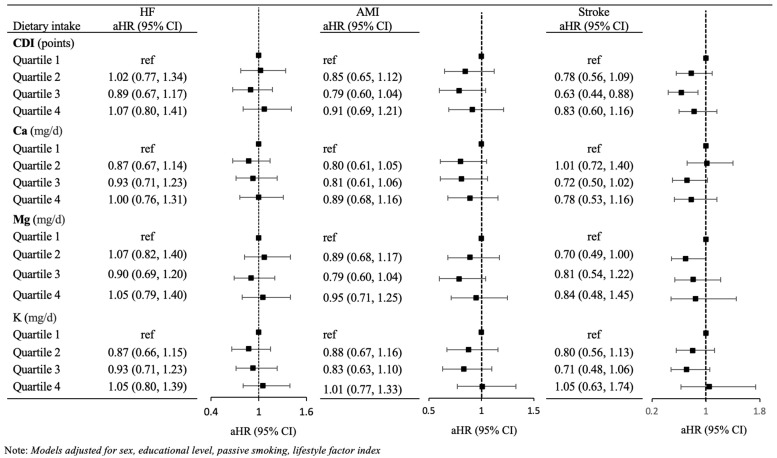
Adjusted hazard ratio (HR) for CVD outcomes per quartile of dietary intake, for combined dietary intake (CDI) score and for individual dietary minerals. HF: Heart Failure; AMI: Acute Myocardial Infarction.

There was little evidence of a consistent, monotonic, protective association between CDI score and risk for each CVD outcome, and confidence intervals were generally wide and included the null hypothesis of no association (Figure 1). However, the third quartile of CDI, compared to the first quartile, was consistently associated with lower risk of AMI (HR: 0.79 [95% CI: 0.60, 1.04]), stroke (HR:0.63 [95% CI: 0.44, 0.88]) and HF (HR:0.89 [95% CI: 0.67, 1.17]). 

Results were similar when considering individual minerals rather than the composite CDI score (Figure 1). Specifically, we did not find evidence of consistent, monotonic associations between any individual mineral and any outcome, but we did observe some lower risk of AMI or stroke associated with the second or third quartile of dietary intake of each mineral. For example, the lowest risk of stroke was associated with the third quartile of Ca (HR: 0.72 [95% CI: 0.50, 1.02]), the second quartile of Mg (0.70 [0.49, 1.00]), and the third quartile of K (0.71 [0.48, 1.06]). However, confidence intervals were wide and consistently included the null hypothesis of no association. 

### 3.2. Secondary Analyses 

**Figure 2:**
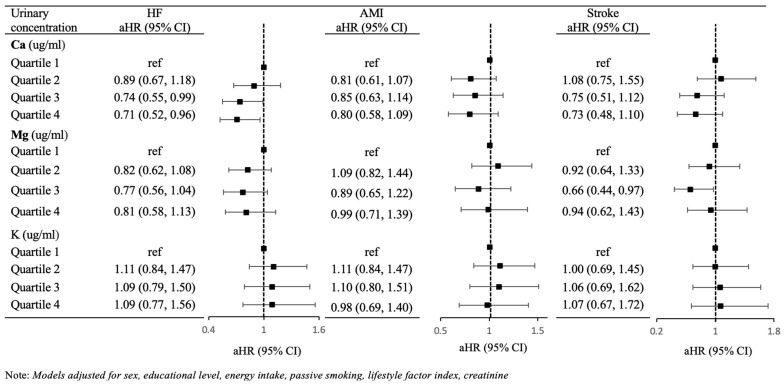
Adjusted hazard ratio (HR) for CVD outcomes per quartile of creatinine-adjusted urinary mineral concentrations. HF: Heart Failure; AMI: Acute Myocardial Infarction.

Results were broadly similar when considering urinary concentrations rather than dietary intake of these minerals (Figure 2), as we did not find evidence of consistent, monotonic associations between urinary concentrations of any individual mineral and CVD outcome. Yet, we did find some isolated associations. For example, the third quartile of Mg was associated with lower risk of stroke (HR: 0.66 [95% CI: 0.44, 0.97]) and heart failure (0.77 [0.56, 1.04]), and the third quartile of Ca was associated with lower risk of heart failure (0.74 [0.55, 0.99]). Again, confidence intervals were wide and generally included the null hypothesis of no association. 

### 3.3. Sensitivity Analyses

Associations between the combined total intake (CTI) score, which included dietary supplements, and each CVD outcome were comparable to the main analyses: there was no clear pattern of monotonic protective associations. Quartile 3 of the CTI score tended to be associated with the lowest risk of each CVD event, reaching statistical significance for heart failure (Table S2). Results of sex-stratified analyses generally followed a similar pattern, with some slight differences observed between males and females (Table S3A,B). Sensitivity analyses for urinary mineral biomarker models were not meaningfully different when evaluating creatinine standardized rather than creatinine adjusted results (Table S4). Within other sensitivity analyses for the main models of CDI score, effect estimates for models restricting on cotinine level and by baseline diagnosis of hypercholesterolemia, diabetes, and hypertension were also comparable to the main findings (Table S5). For stroke, a protective effect was observed among post-menopausal women (HR: 0.47, 95% CI: 0.24, 0.88) when comparing the highest and lowest CDI quartiles (Table S5). Results with and without LFI or LFI components and models adjusting for whole grain and multivitamin supplement use were similar to the main findings (Table S6). Associations for the composite AMI-Stroke outcome were similar to individual AMI and stroke findings (Figure S1). 

## 4. Discussion

Among never-smokers in this Danish population, we did not find evidence to suggest that higher dietary intakes of essential minerals were consistently associated with incident HF, AMI, or stroke. However, the third quartile of CDI was consistently associated with a lower risk of HF, AMI and stroke compared to the first quartile of CDI, potentially suggesting a U-shaped pattern between CDI score and risk of these CVD events, though only associations for stroke reached statistical significance and confidence intervals otherwise remained wide. There are several important contributions from our study design: this is the first study to consider dietary and urinary markers of minerals in relation to HF, AMI, and stroke; we explicitly investigate a combined dietary intake score as a possible improved metric of mineral intake [29,30]; and we also restrict the study population to never smokers to eliminate potential confounding by cigarette smoking. 

### 4.1. Essential Minerals (Ca, Mg, K) and Cardiovascular Events

The results of prior studies of essential minerals (Ca, Mg, K) and CVD outcomes have been inconsistent. When evaluating Ca, for instance, a meta-analysis of 10 prospective cohort studies and approximately 370,000 participants observed inconclusive evidence of association between dietary Ca and stroke, depending on the study subgroup, calcium intake type, and number of years of follow-up [19]. In addition to evaluating dietary calcium intake on the whole, five of these studies more specifically evaluated dairy calcium intake and risk of stroke [54,55,56,57,58]. A meta-analysis of four randomized trials and twenty-seven observational studies found no association between Ca levels in healthy adults and cardiovascular disease (CVD) events and found inconsistent associations with stroke [17]. Another meta-analysis observed no association between Ca supplementation, regardless of concurrent Vitamin D supplementation, on coronary heart disease (CHD) (pooled RR = 1.02, 95% CI: 0.96–1.09) among adult women [20]. In contrast, meta-analyses of 28,072 participants in eight randomized trials of calcium supplements with complete trial-level data found that Ca supplementation, regardless of simultaneous Vitamin D supplementation, increased risk for both MI (pooled RR = 1.24, 95% CI: 1.07–1.45) and stroke (pooled RR = 1.15, 95% CI: 1.00–1.32) [24,25]. The weight of evidence points to potential increased CVD risk from controlled trials of Ca supplementation and null associations with dietary Ca intake, similar to the null association reported here. Still, this prior work did not specifically evaluate populations of never-smokers, and urine Ca was not considered as another indicator of exposure.

Findings from numerous Mg analyses have supported beneficial associations between Mg and CVD [14,21], specifically for HF, stroke, and hypertension. For instance, a recent meta-analysis of forty prospective cohort studies with more than one million participants [22] reported a 7% reduction in stroke risk (RR: 0.93, 95% CI: 0.89–0.97) per 100 mg/day increment of Mg. In the same meta-analysis, Fang et al. also reported negative associations for HF when comparing the highest vs. lowest categories of Mg (RR: 0.69; 95% CI: 0.52, 0.91) and found marginal associations with CHD risk for the highest categories of Mg (RR: 0.90; 95% CI, 0.80, 0.99) [22]. In addition, a meta-analysis of 400,000 adults across eight independent studies observed a 14% (HR: 0.86; 95% CI, 0.81, 0.91) reduction in CV death with higher levels of Mg [59]. In contrast, a meta-analysis of more than 200,000 participants observed no differences in the total risk of CV death when comparing the highest vs. lowest Mg categories [60]. In our study with similar distributions of dietary Mg to those seen in the aforementioned studies, we report significant and nonsignificant risk reduction in CVD outcomes for specific upper quartiles of dietary intake of Mg when compared to the first quartile (HR range: 0.70–1.07), similar to the range of effect estimates reported in the aforementioned meta-analyses (HR range: 0.69–0.90).

When evaluating the literature on K and CVD, higher K levels have been observed to be associated with a lower risk of hypertension [8,61]. Numerous studies have also reported associations between K deficiency and higher ventricular arrhythmia risk among patients who had experienced acute myocardial infarction [62,63,64,65,66,67]. A meta-analysis of 11 studies with nearly 250,000 participants found that an increase of 1.64 g per day of K was associated with a reduction in stroke risk (RR: 0.79; 95% CI: 0.68 to 0.90) and a suggestive reduction in the risk of CHD and total CVD [68]. One systematic review and meta-analysis of 22 randomized controlled trials with 1600 participants observed similar associations, where greater K intake was associated with a lower risk of stroke [8]. Important to note, however, is that K levels in our study were higher than those reported in most studies included in the meta-analyses. In our study, the upper quartiles of K dietary intake (>3800 mg/day; HR range: 0.71–1.05) were not consistently associated with a lower risk of cardiovascular disease, perhaps because our lower quartile of K intake (<3200 mg/day) was at a higher value in comparison to the bulk of the literature. Effect estimates for the third quartile of K, nonetheless, demonstrated a nonsignificant lower incidence of AMI (HR: 0.83, 95% CI: 0.63, 1.10) and stroke (HR: 0.71, 95% CI: 0.48, 1.06) when compared to quartiles 2 and 4.

Two prior studies used a combined dietary intake score for Ca, Mg and K and observed negative associations with stroke for both men and women [29,30]. Distributions of Ca and Mg were similar to those observed in our study, but K was a little higher in our study. In a pooled analysis among women from the Nurses’ Health Study (NHS) I and II, women in the highest vs. lowest quintiles of the combined dietary score had a reduction in the risk of total stroke (0.72, 95% CI: 0.65, 0.81), ischemic stroke (0.78, 95% CI: 0.66, 0.92), and hemorrhagic stroke (0.80, 95% CI: 0.61, 1.04) [29]. In a similar study among men from the Health Professionals Follow-up Study, participants in the highest quintile for a combined dietary score of Mg, K, and Ca had a lower relative risk of total stroke (RR: 0.79, 95% CI: 0.67, 0.92) compared with those in the lowest quintile of combined dietary intake [30]. It was hypothesized that the combined dietary intake scores better captured joint dietary exposures, resulting in a more protective association than was seen from individual mineral analyses. Collectively, we did not see stronger associations using the combined dietary score in our study, although the HR for the third CDI quartile and stroke demonstrated the strongest association (when compared to quartiles 2 and 4) and was significantly protective (HR = 0.63; 95% CI: 0.44, 0.88). 

We did not find a consistent protective association between CDI score and risk for each CVD outcome, as we had hypothesized. Importantly, there may be variations in health responses related to the levels of essential minerals that are evaluated, which may explain some of the lack of consistency in our results. Previous studies have observed the health-protective effects of essential minerals within a specified range, effects which may not be observed at levels approaching nutritional deficiency or nutritional excess and which may create a U-shaped pattern [69,70,71]. Epidemiologic studies, in particular meta-analyses, have begun to report U-shaped associations between dietary Ca and K in relation to cardiovascular outcomes and cardiovascular mortality [72,73,74,75]. These findings may indicate that the difference in mineral intake levels across studies can help to explain inconsistencies in prior literature. In our study, median dietary intake ranged from 1009–1137 mg/d for Ca, 376–384 mg/d for Mg and 3839–3987 mg/d for K (Table 2). Though we did not find evidence to suggest that higher levels of essential minerals are consistently associated with CVD outcomes and found patterns of lower risk of CVD events among the third quartile of CDI, potentially suggesting a U-shaped relationship between CDI score and risk of HF, AMI and stroke, confidence intervals generally remained wide and indicated uncertainty. Upon observing some potential non-linear associations, we performed a posthoc analysis using restricted cubic splines with three knots to more flexibly model associations and to provide more context for these findings (Figures S2–S4). Spline models suggested non-linearity for some of our models, though confidence intervals also indicated uncertainty (Figures S2–S4). Therefore, future work may gain from considering the potential for non-linear associations between dietary minerals and CVD outcomes *a priori*, as well as the range of dietary intake among the population when evaluating the relationships between dietary mineral intake and CVD.

### 4.2. Dietary Intake and Urinary Levels of Minerals and Essential Minerals

Our study collected data on both dietary intake, and for a secondary analysis, urinary levels of essential minerals (Ca, Mg, K). Urinary measures and dietary intake measures were not highly correlated among our study population. Notably, there are key benefits as well as limitations for urinary biomarker measurements vs. dietary intake measurements that may contribute to a lower correlation of these measures in our study. The benefits of urinary biomarkers, for instance, are that they can represent an integrated measure of exposure from multiple exposure routes. However, urinary measures do not account for total excretion since there can be multiple excretion pathways for these minerals beyond urinary excretion. Other limitations of urinary measures are that they may not be able to accurately reflect mineral intake when essential minerals are tightly regulated by the body and are influenced by physiological regulatory factors that impact absorption and excretion parameters [16,76,77,78]. These physiological regulatory factors may call into question the reliability of single-spot urine samples as markers of essential minerals like Ca, Mg and K. 

The benefits of dietary estimates of essential minerals, in contrast, are that these measures are known to correlate with true intake. We used food frequency questionnaires (FFQs) to measure dietary intake. The benefits of the FFQ are that it is a common method for evaluating dietary intake and intake of essential minerals in population-based epidemiology studies [14] and is also a non-invasive tool that is cost-effective and relatively easy to administer. Given the possibility of limitations for reporter bias and within-person variation with FFQs [79,80], we validated the questionnaire [34,81]. FFQ measures are also more interpretable for public health messaging than urinary mineral measures and can allow for concrete health recommendations on dietary intake. 

Additionally, though results were generally similar when considering urinary concentrations rather than dietary intake of minerals, some differences in associations between CVD outcomes and dietary vs. urinary mineral measurements might be expected, given the properties of mineral absorption, distribution, metabolism and excretion in the body. Ca absorption and excretion are closely regulated by a balance of kidney filtration and reabsorption from the renal tubules, as maintaining the level of Ca within a narrow range is important for critical functions [82]. Ca absorption from dietary intake depends on the dose of Ca per day, where approximately 45% of Ca is absorbed from dietary intake at 200 mg Ca/day, but absorption is reduced to 15% when dietary intake reaches 2000 mg Ca/day [78]. The main routes of excretion for Ca are urine and feces, with sweat as a minor route of excretion. Among healthy individuals, urinary Ca increases about 8% as dietary Ca intake increases [76], giving some indication that urinary Ca may reflect dietary exposure. For Mg, about 30–40% of dietary intake is absorbed by the body, with some fluctuation based on the amount of Mg ingested [77]. The primary excretion routes for Mg are urine and feces [83]. Since most Mg in the body is contained within cells or bone [77], evaluating Mg status in the body through biomarkers is difficult. Thus, biomarkers of serum and urine tend to have a low correlation with total body Mg concentrations, and there is no consensus on a gold standard biomarker for Mg [84]. For K, approximately 90% of ingested K is absorbed and used to sustain cellular activity [77]. K is excreted primarily through urine, and excretion increases in response to ingestion of K among healthy individuals [85,86]. Generally, dietary estimates of essential minerals are better known to correlate with true intake, whereas urinary essential mineral measures are less well understood.

### 4.3. Strengths and Limitations

Limitations of our study include the potential for selection bias that could occur if DCH participants were simultaneously more likely than the general population to be exposed to essential minerals and more likely to be a healthier population based on, for example, their socio-demographic status. DCH participants were more likely to be married, have more years of education and have higher income when compared with individuals who did not participate in this study [34]. In sensitivity analyses stratified by education, we did not see substantive differences across education groups and the main findings in Figure 1. By excluding cancer cases from recruitment into the DCH cohort, there might be additional selection bias of a healthier population, which could potentially impact the association between mineral intake and CVD outcomes reported here. Additionally, our reliance on diagnosis codes for ascertainment of HF, AMI and stroke could potentially contribute to some outcome misclassification. Stroke cases were adjudicated, but AMI cases were only adjudicated through 2003 because AMI diagnoses have a positive predictive value greater than 90% in the Danish NPR [87]. HF cases had a positive predictive value of 84% in the Danish NPR [88,89]; these high positive predictive values should minimize the potential for bias due to misclassification of the outcome [90].

Our analytic sample was deliberately restricted to individuals reporting never smoking. This design choice substantially reduces concerns about potential confounding by smoking but also may limit the generalizability of our results to never-smokers. Additionally, the relatively homogenous white population of the DCH study may further limit the generalizability of our results to homogeneous populations in other countries but may also limit potential unmeasured confounding. Discrete time models were also considered in addition to Cox Proportional Hazards models for our analysis; however, the case-cohort design requires a specific code for assigning weights to the sub-cohort to generate estimates of relative risks, which is not available with discrete time models. Therefore, Cox Proportional Hazards models are considered the best approach based on the current literature and methods available. Notably, the follow-up periods for different CVD outcomes (AMI, stroke, HF) in our study vary (e.g., 18.8 years for AMI, 13.6 years for stroke, 19.8 years for HF). These differences in follow-up duration may introduce bias in the comparison of incidence rates and hazard ratios when comparing trends across three outcomes. However, our focus is on comparing results for each outcome with the existing literature, which has variable follow-up times. 

Potential limitations that may lead to a lower correlation between dietary intake and urinary measures in our study could include the timing of specimen collection, as spot urine samples were collected at a single point in time at baseline and could be reflecting a different time period of exposure than dietary intake measures at baseline. Urinary levels may also be impacted by absorption and excretion rates related to other health conditions, such as osteoporosis, and might, therefore, not correlate with daily dietary intake. Similarly, dietary intake and urinary levels measured at baseline may not reflect intake or levels during other time periods, leading to low reproducibility and exposure misclassification. However, an FFQ meta-analysis reported that ICCs for Ca, K and Mg were greater than 0.60 with more than 6 months between FFQ intervals and were considered a reliable instrument of measured dietary intake [91]. Future work may also benefit from greater precision with multiple FFQ measures over time. 

Strengths of our study include the prospective design, with a population-based cohort that allowed for follow-up from baseline (1993–1997) through 2015 and prospectively collected data on dietary intake and urinary concentrations of minerals, HF, AMI, stroke and covariates. This design allowed us to confirm that participants were not diagnosed with the outcome of interest at study start. Urinary samples and food frequency data were also collected before outcome diagnosis with this prospective design. To our knowledge, this study is among the first to evaluate both mineral dietary intake and urinary mineral levels of Ca, Mg and K in association with HF, AMI and stroke. Our work adds to the current literature by having a moderately large sample size to investigate the prospective risk of HF, AMI and stroke among never-smokers. 

## 5. Conclusions

We did not find consistent evidence to suggest that a higher intake of essential minerals is associated with incident HF, AMI or stroke. We observed a pattern of results suggesting a potential U-shaped relationship when evaluating combined intake of essential minerals and CVD outcomes, though confidence intervals were generally wide; this U-shaped relationship should be explicitly considered *a priori* in future work on dietary minerals and CVD.

## Figures and Tables

**Table 1:** Baseline characteristics for a subset of never smoking DCH participants in a CVD outcomes case–cohort study.

Baseline Characteristics	Subcohort (n = 1135)	Heart Failure Cases (n = 893)	AMI Cases (n = 776)	Stroke Cases (n = 502)	*p*-Value
	Mean (SD) or %	Mean (SD) or %	Mean (SD) or %	Mean (SD) or %	
Age at enrolment (years)	56.4 (4.3)	58.2 (4.3)	57.9 (4.2)	58.0 (4.41)	<0.001
Gender					
*Female*	49.6	49.4	39.6	47.8	<0.001
*Male*	50.4	50.6	60.4	52.2	
Marital status					
*Married*	75.4	66.6	71.5	71.1	0.005
*Divorced*	12.0	15.5	14.3	13.9	
*Widowed*	5.0	7.7	6.6	5.6	
*Unmarried*	6.9	9.6	6.4	8.0	
*Unanswered*	0.8	0.4	1.2	1.4	
Employed	81.2	74.1	76.0	71.7	<0.001
Years in School					
*Low (≤7 years)*	28.3	36.4	32.5	34.9	0.004
*Medium (8–10 years)*	45.8	43.9	47.6	41.6	
*High (> 10 years)*	25.9	19.7	20.0	23.5	
BMI (kg/m^3^)	26.4 (4.2)	26.8 (5.1)	27.4 (4.2)	27.4 (4.6)	<0.001
Used Dietary Supplement	72.7	66.5	65.7	67.3	0.003
Self-reported hypertension	16.7	11.9	27.2	31.3	<0.001
Urine Cotinine (µg/L)	45.1 (112.6)	37.0 (99.1)	35.2 (90.3)	32.8 (185.9)	0.294
Lifestyle Factor Index (LFI)					
*0*	0.0	0.0	0.0	0.0	<0.001
*1*	1.2	1.3	1.7	1.6	
*2*	25.2	41.9	32.4	36.5	
*3*	69.1	53.6	61.3	57.6	
*4*	4.5	3.13	4.6	4.4	
Parity (Women)					
*0*	12.6	14.1	10.4	13.8	0.840
*1*	11.7	11.1	12.7	12.1	
*2*	47.6	43.3	47.2	46.7	
*3+*	28.1	31.5	29.6	27.5	
Post-menopausal Status (Women)	81.9	92.1	90.6	90.0	0.001

For the Lifestyle Factor Index (LFI), 0 indicates less healthy lifestyle factors, and 4 indicates healthier lifestyle factors. *p*-values reported from one-way ANOVA for “Age at enrolment”, “Body Mass Index” and “Urine Cotinine”, with others through chi-square (x^2^) tests.

**Table 2:** Dietary intake of minerals (mg/d) and creatinine standardized urinary concentrations of minerals (mg/g creatinine).

	Subcohort (n = 1135)		Heart Failure Cases (n = 893)		AMI Cases (n = 776)		Stroke Cases (n = 502)	
	Median (SD)	IQR (Q1, Q3)	Median (SD)	IQR (Q1, Q3)	Median (SD)	IQR (Q1, Q3)	Median (SD)	IQR (Q1, Q3)
Combined Dietary Intake (CDI) score (points) *								
7.2 (1.14)		(5.0, 10.0)	7.0 (1.12)	(5.0, 10.0)	8.0 (3.0)	(5.0, 10.0)	7.50 (3.0)	(5.0, 10.0)
Dietary Intake (mg/d)								
Ca	1068.7 (447.9)	(802.2, 1377.7)	1136.80 (461.87)	(798.80, 1397.80)	1063.1 (467.7)	(792.9, 1391.7)	1008.8 (462.2)	(782.0, 1340.3)
Mg	375.6 (94.4)	(318.9, 439.7)	383.70 (98.03)	(316.70, 440.50)	375.6 (96.9)	(318.0, 443.0)	382.1 (104.3)	(308.3, 438.2)
K	3894.0 (1003.1)	(3266.0, 4543.0)	3986.50 (1059.07)	(3253.20, 4583.30)	3904 (1044.8)	(3282.4, 4598.4)	3839.0 (1129.0)	(3191.0, 4620.0)
Urinary Biomarkers (mg/g creatinine)								
Ca	120.4 (156.0)	(73.8, 196.1)	116.1 (150.4)	(70.7, 189.5)	117.5 (159.0)	(70.7, 191.0)	149.6 (152.5)	(66.5, 173.3)
Mg	65.4 (68.1)	(44.9, 96.8)	64.4 (73.0)	(44.2, 95.3)	63.3 (67.9)	(43.9, 94.4)	80.4 (82.9)	(41.5, 87.9)
K	2244.5 (1751.3	(1563.1, 3240.0)	2236.8 (1847.5)	(1561.8, 3209.8)	2210.6 (1701.8)	(1539.5, 3213.2)	2633.0 (1676.3)	(1560.9, 3170.6)

* Combined dietary intake (CDI) score based on joint Ca, Mg and K intakes (mg/d) from the FFQ. Participants with levels in the highest quartile for each mineral (Ca, Mg and K) received a score of 4 points for each mineral, for a total of 12 points (maximum CDI score). Accordingly, the CDI score ranged from 3–12 points.

## Data Availability

The data presented in this study are available on request from the corresponding author due to (specify the reason for the restriction).

## Supplementary Materials

The following supporting information can be downloaded at https://www.mdpi.com/article/10.3390/ijerph21070932/s1, Table S1: Spearman correlation coefficient matrix for correlations between dietary and creatinine-adjusted urinary measures of minerals^a^; Table S2. Including Dietary Supplements: Adjusted hazard ratio (HR) for CVD outcomes per quartile of combined total intake (CTI) score (dietary intake and supplement intake dose) and per total individual intake level, Table S3A: Adjusted hazard ratio (aHR) for CVD outcomes per quartile of the combined dietary score and per quartile of creatinine adjusted urinary mineral concentrations (sex-stratified); Table S3B: Adjusted hazard ratio (aHR) for composite AMI-Stroke outcome per quartile of the combined dietary score and per quartile of creatinine adjusted urinary mineral concentrations (sex-stratified); Table S4: Adjusted hazard ratio (HR) for CVD outcomes per quartile of urinary mineral concentrations, creatinine-standardized and creatinine adjusted models; Table S5: Adjusted hazard ratio (HR) for CVD outcomes per quartile of dietary intake concentrations, with restrictions; Table S6. Adjusted hazard ratio (HR) for CVD outcomes per quartile of combined dietary score, by covariate models Figure S1. Adjusted hazard ratio (HR) for Composite AMI-Stroke outcomes per quartile dietary intake and urinary concentration of minerals; Figure S2. Splines for dietary mineral intake (mg/day) of calcium, magnesium and potassium and heart failure (HF); Figure S3. Splines for dietary mineral intake (mg/day) of calcium, magnesium and potassium and acute myocardial infarction (AMI); Figure S4. Splines for dietary mineral intake (mg/day) of calcium, magnesium and potassium and stroke.
