# Supplementary material for: Dietary Minerals and Incident Cardiovascular Outcomes among Never-Smokers in a Danish Case–Cohort Study
Source: Int J Environ Res Public Health. 2024 Jul 17;21(7):932. doi: 10.3390/ijerph21070932 (PMC11277515; doi:10.3390/ijerph21070932)

SUPPLEMENTAL MATERIAL

Dietary Minerals and Incident Cardiovascular Outcomes among Never-Smokers in a Danish Case–Cohort Study

Table S1. Spearman correlation coefficient matrix for correlations between dietary and creatinine-adjusted urinary measures of minerals[a]

	Dietary K	Dietary Mg	Dietary Ca	Urinary K	Urinary Mg	Urinary Ca
	**Heart Failure**					
Dietary K	1.000					
Dietary Mg	0.93	1.000				
Dietary Ca	0.71	0.71	1.000			
Urinary K	0.01	-0.02	-0.04	1.000		
Urinary Mg	-0.01	-0.02	-0.03	0.44	1.000	
Urinary Ca	-0.03	-0.05	-0.03	0.32	0.57	1.000
	**AMI**					
Dietary K	1.000					
Dietary Mg	0.91	1.000				
Dietary Ca	0.68	0.68	1.000			
Urinary K	0.07	0.04	0.09	1.000		
Urinary Mg	-0.03	-0.03	0.05	0.33	1.000	
Urinary Ca	-0.02	-0.02	0.07	0.29	0.63	1.000
	**Stroke**					
Dietary K	1.000					
Dietary Mg	0.93	1.000				
Dietary Ca	0.71	0.71	1.000			
Urinary K	0.04	0.01	-0.01	1.000		
Urinary Mg	0.001	-0.002	-0.03	0.45	1.000	
Urinary Ca	-0.02	-0.03	-0.02	0.32	0.61	1.000

[a]K=potassium; Mg = magnesium; Ca = calcium;

Table S2. Including Dietary Supplements: Adjusted hazard ratio (HR) for CVD outcomes per quartile of combined total intake (CTI) score (dietary intake and supplement intake dose) and per total individual intake level[a]

	aHR (95% CI)			
	Heart Failure (n=1969)	AMI (n=1855)	Stroke (n=1594)	Composite AMI-Stroke Outcome (n=2276)
**Combined total intake score (CTI) (points)**				
Quartile 1	ref	ref	ref	ref
Quartile 2	0.86 (0.65, 1.13)	0.89 (0.68, 1.17)	0.88 (0.63, 1.22)	0.85 (0.68, 1.07)
Quartile 3	0.64 (0.47, 0.88)	0.84 (0.64, 1.11)	0.74 (0.52, 1.04)	0.80 (0.63, 1.01)
Quartile 4	0.75 (0.53, 1.05)	0.95 (0.72, 1.25)	0.83 (0.60, 1.16)	0.90 (0.71, 1.13)
**Ca[a] (mg/d)**				
Quartile 1	ref	ref	ref	ref
Quartile 2	0.96 (0.73, 1.25)	0.85 (0.65, 1.12)	1.06 (0.77, 1.44)	0.95 (0.76, 1.20)
Quartile 3	0.93 (0.71, 1.23)	0.86 (0.66, 1.13)	0.85 (0.62, 1.18)	0.90 (0.71, 1.13)
Quartile 4	1.02 (0.78, 1.34)	0.88 (0.67, 1.16)	0.84 (0.60, 1.16)	0.91 (0.72, 1.14)
**Mg[a] (mg/d)**				
Quartile 1	ref	ref	ref	ref
Quartile 2	1.01 (0.77, 1.33)	0.98 (0.75, 1.29)	0.85 (0.62, 1.17)	0.94 (0.75, 1.18)
Quartile 3	0.87 (0.66, 1.15)	0.87 (0.66, 1.15)	0.79 (0.57, 1.10)	0.90 (0.71, 1.15)
Quartile 4	1.03 (0.78, 1.36)	0.96 (0.73, 1.26)	0.78 (0.56, 1.09)	0.92 (0.73, 1.17)
**K[a] (mg/d)**				
Quartile 1	ref	ref	ref	ref
Quartile 2	0.78 (0.66, 1.15)	0.89 (0.68, 1.17)	0.75 (0.55, 1.03)	0.92 (0.73, 1.15)
Quartile 3	0.93 (0.70, 1.22)	0.83 (0.63, 1.09)	0.66 (0.47, 0.91)	0.88 (0.70, 1.10)
Quartile 4	1.06 (0.80, 1.41)	1.01 (0.77, 1.33)	0.86 (0.62, 1.19)	0.99 (0.78, 1.26)

Note: Models adjusted for gender, education level, passive smoking, lifestyle factor index.

[a]Total individual intake (mg/d): summing dietary intake mg/d and supplemental intake (mg/d) for each mineral

Table S3A: Adjusted hazard ratio (aHR) for CVD outcomes per quartile of the combined dietary score and per quartile of creatinine adjusted urinary mineral concentrations (sex-stratified)

	WOMEN			MEN		
	aHR (95% CI)			aHR (95% CI)		
	Heart Failure (n=982)	AMI (n=857)	Stroke (n=790)	Heart Failure (n=987)	AMI (n=998)	Stroke (n=804)
**Combined dietary intake score (points)**[a]						
Quartile 1	ref	ref	ref	ref	ref	ref
Quartile 2	0.73 (0.50, 1.07)	0.96 (0.66, 1.40)	0.82 (0.54, 1.25)	0.93 (0.60, 1.46)	0.76 (0.50, 1.15)	0.79 (0.48, 1.32)
Quartile 3	0.72 (0.48, 1.06)	0.68 (0.46, 1.00)	0.60 (0.39, 0.94)	0.85 0.54, 1.34)	0.82 (0.54, 1.24)	0.66 (0.39, 1.09)
Quartile 4	0.94 (0.64, 1.39)	0.95 (0.62, 1.45)	0.72 (0.45, 1.16)	0.91 (0.59, 1.41)	0.85 (0.57, 1.27)	0.90 (0.56, 1.46)
**Urinary Measures**[b]						
**Ca** (µg/ml)						
Quartile 1	ref	ref	ref	ref	ref	ref
Quartile 2	0.85 (0.58, 1.24)	1.07 (0.72, 1.60)	0.90 (0.58, 1.39)	0.90 (0.59, 1.38)	0.74 (0.51, 1.07)	0.82 (0.52, 1.28)
Quartile 3	0.70 (0.67, 1.05)	0.65 (0.43, 0.97)	0.53 (0.34, 0.84)	0.75 (0.48, 1.16)	0.75 (0.52, 1.08)	0.69 (0.44, 1.10)
Quartile 4	0.80 (0.51, 1.26)	0.79 (0.53, 1.20)	0.75 (0.48, 1.18)	0.64 (0.40, 1.00)	0.80 (0.55, 2.25)	0.70 (0.44, 1.10)
**Mg** (µg/ml)						
Quartile 1	ref	ref	ref	ref	ref	ref
Quartile 2	0.84 (0.57, 1.21)	1.07 (0.72, 1.60)	0.73 (0.44, 1.22)	0.74 (0.48, 1.14)	0.76 (0.53, 1.09)	1.12 (0.72, 1.75)
Quartile 3	0.90 (0.59, 1.36)	0.70 (0.46, 1.05)	0.86 (0.54, 1.37)	0.60 (0.38, 0.97)	0.83 (0.58, 1.20)	0.69 (0.44, 1.10)
Quartile 4	0.87 (0.55, 1.36)	0.78 (0.52, 1.17)	0.62 (0.39, 0.97)	0.71 (0.43, 1.18)	1.07 (0.75, 1.54)	0.97 (0.62, 1.53)
**K** (µg/ml)						
Quartile 1	ref	ref	ref	ref	ref	ref
Quartile 2	1.08 (0.74, 1.57)	0.77 (0.51, 1.15)	1.06 (0.68, 1.66)	1.11 (0.71, 1.75)	0.73 (0.51, 1.05)	0.94 (0.60, 1.49)
Quartile 3	1.34 (0.87, 2.08)	0.73 (0.48, 1.09)	0.92 (0.58, 1.45)	0.92 (0.57, 1.50)	1.07 (0.75, 1.53)	1.01 (0.64, 1.60)
Quartile 4	0.98 (0.60, 1.61)	0.86 (0.58, 1.28)	0.74 (0.60, 1.48)	1.20 (0.70, 2.04)	1.04 (0.73, 1.50)	0.93 (0.59, 1.48)

[a]Models adjusted for sex, education level, passive smoking, lifestyle factor index

[b] Models adjusted for sex, education level, energy intake, passive smoking, lifestyle factor, creatinine

Table S3B: Adjusted hazard ratio (aHR) for composite AMI-Stroke outcome per quartile of the combined dietary score and per quartile of creatinine adjusted urinary mineral concentrations (sex-stratified)

	**WOMEN** (n=1076)	**MEN** (n=1200)
	aHR (95% CI) Combined AMI-Stroke Outcome	
**Combined dietary intake score (points)**[a]		
Quartile 1	ref	ref
Quartile 2	0.93 (0.68, 1.30)	0.73 (0.53, 1.03)
Quartile 3	0.79 (0.57, 1.09)	0.79 (0.57, 1.11)
Quartile 4	0.84 (0.61, 1.17)	0.83 (0.60, 1.15)
**Urinary Measures**[b]		
**Ca** (µg/ml)		
Quartile 1	ref	ref
Quartile 2	1.03 (0.71, 1.48)	0.86 (0.645 1.14)
Quartile 3	0.74 (0.52, 1.05)	0.95 (0.71, 1.28)
Quartile 4	0.80 (0.58, 1.10)	0.90 (0.64, 1.26)
**Mg** (µg/ml)		
Quartile 1	ref	ref
Quartile 2	0.93 (0.64, 1.36)	0.86 (0.66, 1.15)
Quartile 3	0.85 (0.60, 1.20)	0.93 (0.70, 1.26)
Quartile 4	0.74 (0.53, 1.04)	1.06 (0.76, 1.50)
**K (**µg/ml)		
Quartile 1	ref	ref
Quartile 2	0.73 (0.50, 1.07)	1.12 (0.85, 1.48)
Quartile 3	0.78 (0.55, 1.12)	1.11 (0.83, 1.50)
Quartile 4	0.83 (0.59, 1.16)	1.08 (0.76, 1.53)

[a]Models adjusted for sex, education level, passive smoking, lifestyle factor index
[b]Models adjusted for sex, education level, energy intake, passive smoking, lifestyle factor index, creatinine

Table S4: Adjusted hazard ratio (HR) for CVD outcomes per quartile of urinary mineral concentrations, creatinine-standardized and creatinine adjusted models

Urinary Minerals	Creatinine standardized model[a] (mg/g creatinine)	Creatinine adjusted model[b] (µg/ml)
	**Stroke**	
**Ca**		
Quartile 1	ref	ref
Quartile 2	0.82 (0.60, 1.12)	1.08 (0.75, 1.55)
Quartile 3	0.70 (0.51, 0.96)	0.75 (0.51, 1.12)
Quartile 4	0.62 (0.45, 0.87)	0.73 (0.48, 1.10)
**Mg**		
Quartile 1	ref	ref
Quartile 2	0.82 (0.60, 1.12)	0.92 (0.64, 1.33)
Quartile 3	0.98 (0.71, 1.34)	0.66 (0.44, 0.97)
Quartile 4	0.74 (0.53, 1.04)	0.94 (0.62, 1.43)
**K**		
Quartile 1	ref	ref
Quartile 2	1.03 (0.75, 1.42)	1.00 (0.69, 1.45)
Quartile 3	1.03 (0.74, 1.42)	1.06 (0.69, 1.62)
Quartile 4	0.95 (0.68, 1.32)	1.07 (0.67, 1.72)
	**AMI**	
**Ca**		
Quartile 1	ref	ref
Quartile 2	0.78 (0.60, 1.02)	0.81 (0.61, 1.07)
Quartile 3	0.79 (0.60, 1.04)	0.85 (0.63, 1.14)
Quartile 4	0.81 (0.62, 1.07)	0.80 (0.58, 1.09)
**Mg**		
Quartile 1	ref	ref
Quartile 2	0.88 (0.67, 1.15)	1.09 (0.82, 1.44)
Quartile 3	0.90 (0.68, 1.18)	0.89 (0.65, 1.22)
Quartile 4	0.92 (0.69, 1.22)	0.99 (0.71, 1.39)
**K**		
Quartile 1	ref	ref
Quartile 2	0.96 (0.73, 1.25)	1.11 (0.84, 1.47)
Quartile 3	0.99 (0.75, 1.29)	1.10 (0.80, 1.51)
Quartile 4	1.01 (0.76, 1.34)	0.98 (0.69, 1.40)
	**Heart Failure**	
**Ca**		
Quartile 1	ref	ref
Quartile 2	0.82 (0.62, 1.07)	0.89 (0.67, 1.18)
Quartile 3	0.67 (0.51, 0.88)	0.74 (0.55, 0.99)
Quartile 4	0.68 (0.51, 0.89)	0.71 (0.52, 0.96)
**Mg**		
Quartile 1	ref	ref
Quartile 2	0.86 (0.66, 1.13)	0.82 (0.62, 1.08)
Quartile 3	0.93 (0.71, 1.22)	0.77 (0.56, 1.04)
Quartile 4	0.91 (0.69, 1.21)	0.81 (0.58, 1.13)
**K**		
Quartile 1	ref	ref
Quartile 2	0.91 (0.69, 1.19)	1.11 (0.84, 1.47)
Quartile 3	1.02 (0.78, 1.34)	1.09 (0.79, 1.50)
Quartile 4	0.90 (0.67, 1.19)	1.09 (0.77, 1.56)

	Composite AMI-Stroke	
**Ca**		
Quartile 1	ref	ref
Quartile 2	0.92 (0.74, 1.13)	0.95 (0.76, 1.20)
Quartile 3	0.86 (0.70, 1.07)	0.94 (0.75, 1.18)
Quartile 4	0.85 (0.69, 1.06)	0.87 (0.69, 1.10)
**Mg**		
Quartile 1	ref	ref
Quartile 2	0.89 (0.71, 1.11)	1.05 (0.83, 1.32)
Quartile 3	0.91 (0.72, 1.14)	0.94 (0.74, 1.18)
Quartile 4	0.87 (0.69, 1.11)	1.05 (0.83, 1.32)
**K**		
Quartile 1	ref	ref
Quartile 2	0.98 (0.79, 1.21)	1.06 (0.85, 1.33)
Quartile 3	0.99 (0.79, 1.22)	1.05 (0.81, 1.35)
Quartile 4	1.00 (0.80, 1.25)	1.04 (0.78, 1.38)

[a] Adjusting for gender, education level, energy intake, passive smoking, lifestyle factor index

[b] Adjusting for gender, education level, energy intake, passive smoking, lifestyle factor index, creatinine

Table S5: Adjusted hazard ratio (HR) for CVD outcomes per quartile of dietary intake concentrations, with restrictions

Combined dietary intake score (CDI) (points)	Total[a]	Restriction: Cotinine Levels (90th percentile: <56.20 ug/L)[a] N= 1723	Restriction: Women, post menopause only[b] N=618	Restriction: Participants without hypercholesterolemia, diabetes, hypertension dx at baseline[a] n=1385
**Heart Failure**				
Quartile 1	ref	ref	ref	ref
Quartile 2	1.02 (0.77, 1.34)	0.98 (0.73, 1.32)	0.97 (0.61, 1.52)	0.91 (0.65, 1.27)
Quartile 3	0.89 (0.67, 1.17)	0.82 (0.61, 1.10)	0.88 (0.55, 1.40)	0.83 (0.59, 1.16)
Quartile 4	1.07 (0.81, 1.41)	1.10 (0.81, 1.48)	0.92 (0.56, 1.51)	0.97 (0.69, 1.36)
**AMI**				
	Total[a]	Restriction: Cotinine Levels (90th percentile: <59.38 ug/L)[a] N= 1540	Restriction: Women, post menopause only[b] N=726	Restriction: Participants without hypercholesterolemia, diabetes, hypertension dx at baseline[a] N=1354
Quartile 1	ref	ref	ref	ref
Quartile 2	0.85 (0.65, 1.12)	0.83 (0.62, 1.13)	1.09 (0.71, 1.67)	0.89 (0.64, 1.25)
Quartile 3	0.79 (0.60, 1.04)	0.76 (0.56, 1.03)	0.66 (0.42, 1.02)	0.81 (0.58, 1.14)
Quartile 4	0.91 (0.69, 1.21)	0.96 (0.70, 1.31)	0.94 (0.61, 1.45)	1.00 (0.72, 1.42)
**Stroke**				
	Total[a]	Restriction: Cotinine Levels (90th percentile: <58.90 ug/L)[a] N= 1434	Restriction: Women, post menopause only[b] N=468	Restriction: Participants without hypercholesterolemia, diabetes, hypertension dx at baseline[a] n=1175
Quartile 1	ref	ref	ref	ref
Quartile 2	0.78 (0.56, 1.09)	0.79 (0.56, 1.11)	0.65 (0.38, 1.12)	0.85 (0.57, 1.25)
Quartile 3	0.63 (0.44, 0.88)	0.61 (0.43, 0.87)	0.65 (0.37, 1.13)	0.64 (0.42, 0.96)
Quartile 4	0.83 (0.60, 1.16)	0.89 (0.63, 1.25)	0.47 (0.24, 0.88)	0.89 (0.60, 1.32)
**Composite AMI-Stroke**				
	Total[a]	Restriction: Cotinine Levels (90th percentile: <62.50 ug/L)[a] N= 1980	Restriction: Women, post menopause only[b] N=924	Restriction: Participants without hypercholesterolemia, diabetes, hypertension dx at baseline[a] n=1614
Quartile 1	ref	ref	ref	ref
Quartile 2	0.85 (0.68, 1.07)	0.90 (0.71, 1.13)	1.12 (0.79, 1.60)	0.89 (0.69, 1.16)
Quartile 3	0.80 (0.63, 1.01)	0.93 (0.74, 1.17)	0.89 (0.62, 1.26)	0.92 (0.70, 1,18)
Quartile 4	0.90 (0.71, 1.13)	0.99 (0.79, 1.25)	0.95 (0.66, 1.36)	1.06 (0.82, 1.37)

[a]Models adjusted for sex, education level, passive smoking, lifestyle factor index; [b]Models adjusted for education level, passive smoking, lifestyle factor index

Table S6. Adjusted hazard ratio (HR) for CVD outcomes per quartile of combined dietary score, by covariate models

**Combined dietary intake score (points)**	Unadjusted Model	Main Model[a]	Model without LFI[b]	Model with LFI components[c]	Model with whole grain[d]	Model with supplement use[e]	Model with whole grain and supplement use[f]
**Heart Failure**							
Quartile 1	ref	ref	ref	ref	ref	ref	ref
Quartile 2	1.02 (0.77, 1.34)	1.01 (0.77, 1.33)	1.00 (0.77, 1.32)	1.08 (0.82, 1.42)	1.01 (0.77, 1.32)	1.07 (0.81, 1.42)	1.02 (0.77, 1.34)
Quartile 3	0.89 (0.67, 1.17)	0.82 (0.63, 1.08)	0.82 (0.63, 1.08)	0.91 (0.69, 1.21)	0.85 (0.65, 1.11)	0.93 (0.70, 1.24)	0.89 (0.67, 1.17)
Quartile 4	1.07 (0.81, 1.41)	1.03 (0.78, 1.35)	1.02 (0.78, 1.35)	1.25 (0.91, 1.71)	1.06 (0.80, 1.40)	1.27 (0.93, 1.75)	1.07 (0.81, 1.41)
**AMI**							
Quartile 1	ref	ref	ref	ref	ref	ref	ref
Quartile 2	0.95 (0.73, 1.24)	0.85 (0.65, 1.12)	0.85 (0.64, 1.12)	0.83 (0.62, 1.12)	0.89 (0.67, 1.18)	0.86 (0.65, 1.14)	0.90 (0.68, 1.19)
Quartile 3	0.84 (0.65, 1.09)	0.79 (0.60, 1.04)	0.76 (0.58, 1.01)	0.77 (0.55, 1.07)	0.85 (0.64, 1.14)	0.81 (0.62, 1.08)	0.88 (0.65, 1.17)
Quartile 4	1.09 (0.83, 1.42)	0.91 (0.69, 1.21)	0.90 (0.68, 1.19)	0.90 (0.59, 1.37)	1.05 (0.77, 1.45)	0.94 (0.71, 1.25)	1.08 (0.78, 1.48)
**Stroke**							
Quartile 1	ref	ref	ref	ref	ref	ref	ref
Quartile 2	0.81 (0.59, 1.11)	0.80 (0.58, 1.10)	0.81 (0.58, 1.11)	0.83 (0.60, 1.15)	0.80 (0.58, 1.10)	0.81 (0.59, 1.13)	0.81 (0.59, 1.11)
Quartile 3	0.63 (0.46, 0.88)	0.61 (0.44, 0.84)	0.63 (0.45, 0.88)	0.66 (0.46, 0.93)	0.65 (0.47, 0.91)	0.67 (0.47, 0.95)	0.63 (0.46, 0.88)
Quartile 4	0.83 (0.60, 1.15)	0.81 (0.59, 1.12)	0.79 (0.57, 1.10)	0.86 (0.59, 1.24)	0.79 (0.57, 1.10)	0.84 (0.57, 1.22)	0.83 (0.60, 1.15)
**Composite AMI-Stroke**							
Quartile 1	ref	ref	ref	ref	ref	ref	ref
Quartile 2	0.90 (0.72, 1.13)	0.85 (0.68, 1.07)	0.90 (0.72, 1.11)	0.91 (0.71, 1.16)	0.92 (0.73, 1.16)	0.91 (0.72, 1.15)	0.92 (0.73, 1.17)
Quartile 3	0.87 (0.70, 1.09)	0.80 (0.63, 1.01)	0.85 (0.69, 1.06)	0.87 (0.66, 1.14)	0.90 (0.71, 1.14)	0.89 (0.70, 1.12)	0.91 (0.71, 1.17)
Quartile 4	0.93 (0.74, 1.16)	0.90 (0.71, 1.13)	0.90 (0.72, 1.11)	0.91 (0.65, 1.27)	0.97 (0.75, 1.26)	0.94 (0.74, 1.19)	0.99 (0.76, 1.29)

Note: LFI=Lifestyle Factor Index;

[a] Adjusting for gender, education level, passive smoking, lifestyle factor index (LFI)

[b] Adjusting for gender, education level, passive smoking

[c] Adjusting for gender, education level, passive smoking, alcohol intake (drinks/week), waist circumference (cm), physical activity at least 30 mins/day (yes/no), dietary criteria (yes/no: >600 g fruits/vegetables per day, ≤ 500 g red/processed meat/week, and ≥ 3 dietary fiber per MJ dietary energy)

[d] Adjusting for gender, education level, passive smoking, alcohol intake (drinks/week), waist circumference (cm), physical activity at least 30 mins/day (yes/no), dietary criteria (yes/no: >600 g fruits/vegetables per day, ≤ 500 g red/processed meat/week, and ≥ 3 dietary fiber per MJ dietary energy), whole grain intake (grams of whole grain cereal)

[e] Adjusting for gender, education level, passive smoking, alcohol intake (drinks/week), waist circumference (cm), physical activity at least 30 mins/day (yes/no), dietary criteria (yes/no: >600 g fruits/vegetables per day, ≤ 500 g red/processed meat/week, and ≥ 3 dietary fiber per MJ dietary energy), supplement use (yes vs. no)

[f] Adjusting for gender, education level, passive smoking, alcohol intake (drinks/week), waist circumference (cm), physical activity at least 30 mins/day (yes/no), dietary criteria (yes/no: >600 g fruits/vegetables per day, ≤ 500 g red/processed meat/week, and ≥ 3 dietary fiber per MJ dietary energy), supplement use (yes vs. no), whole grain intake (grams of whole grain cereal)

Figure S1. Adjusted hazard ratio (HR) for Composite AMI-Stroke outcomes per quartile dietary intake and urinary concentration of minerals

Combined AMI-Stroke Outcomes

Dietary Intake	aHR (95% CI)	Urinary Concentration	aHR (95% CI)
**CDI** (points)			
Quartile 1	ref		
Quartile 2	0.85 (0.68, 1.07)		
Quartile 3	0.80 (0.63, 1.01)		
Quartile 4	0.90 (0.71, 1.13)		
**Ca** (mg/d)		**Ca** (ug/ml)	
Quartile 1	ref	Quartile 1	ref
Quartile 2	0.92 (0.74, 1.16)	Quartile 2	0.95 (0.76, 1.20)
Quartile 3	0.84 (0.67, 1.06)	Quartile 3	0.94 (0.75, 1.18)
Quartile 4	0.90 (0.72, 1.13)	Quartile 4	0.87 (0.69, 1.10)
**Mg** (mg/d)		**Mg** (ug/ml)	
Quartile 1	ref	Quartile 1	ref
Quartile 2	0.91 (0.72, 1.15)	Quartile 2	1.05 (0.83, 1.32)
Quartile 3	0.86 (0.68, 1.08)	Quartile 3	0.94 (0.74, 1.18)
Quartile 4	0.92 (0.73, 1.78)	Quartile 4	1.05 (0.83, 1.32)
K (mg/d)		**K** (ug/ml)	
Quartile 1	ref	Quartile 1	ref
Quartile 2	0.91 (0.73, 1.15)	Quartile 2	1.06 (0.85, 1.33)
Quartile 3	0.88 (0.70, 1.11)	Quartile 3	1.05 (0.81, 1.35)
Quartile 4	0.99 (0.78, 1.25)	Quartile 4	1.04 (0.78, 1.38)

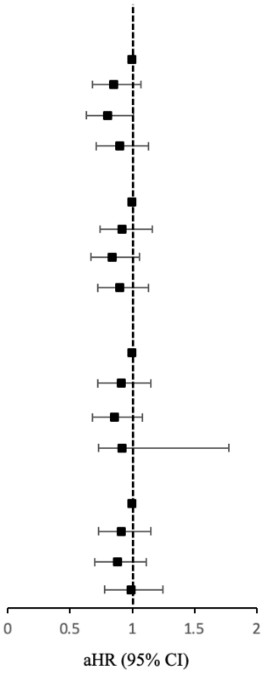

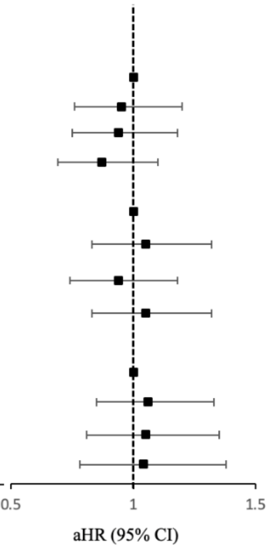

Figure S2. Splines for dietary mineral intake (mg/day) of calcium, magnesium and potassium and heart failure (HF)

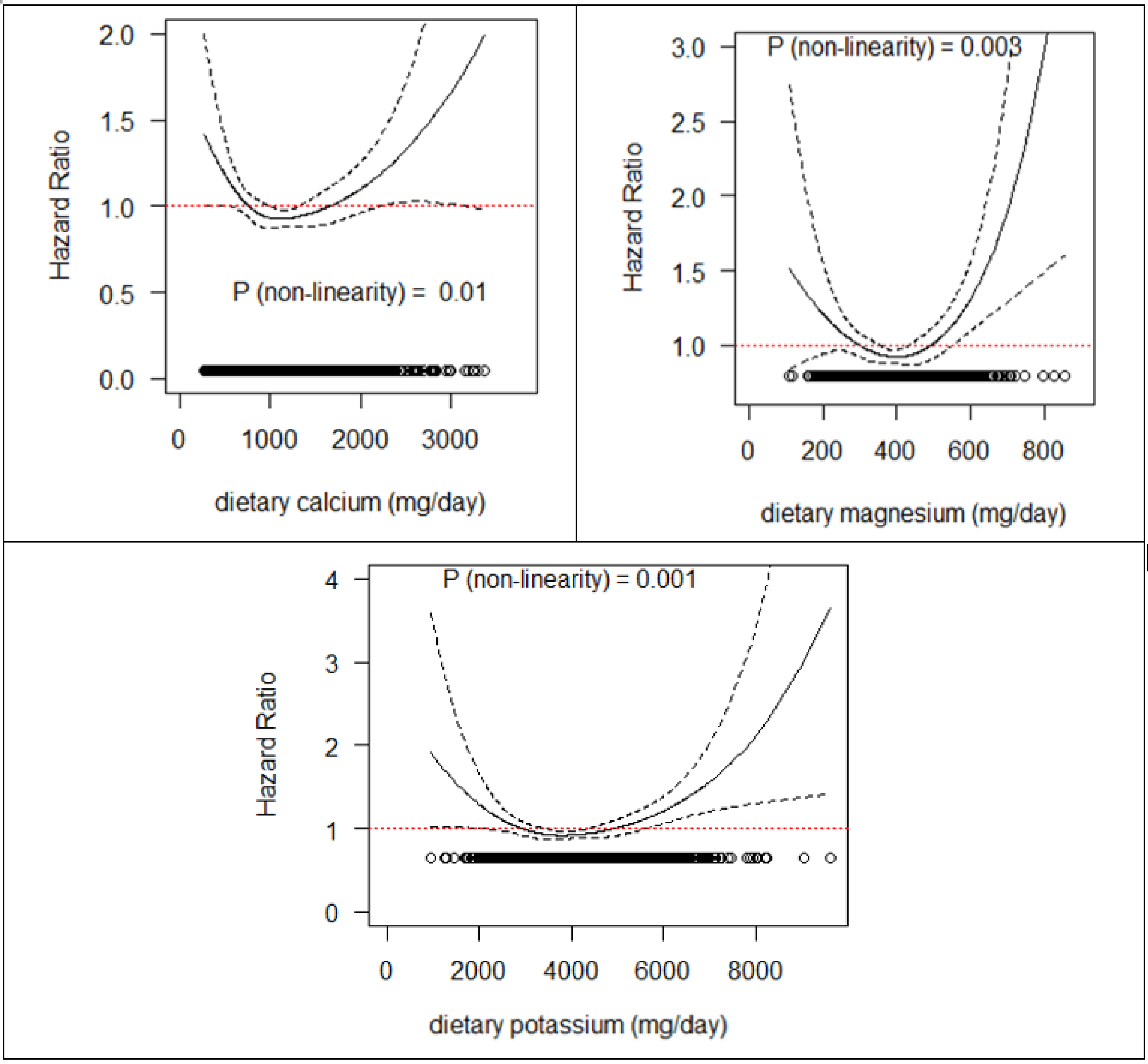

Figure S3. Splines for dietary mineral intake (mg/day) of calcium, magnesium and potassium and acute myocardial infarction (AMI)

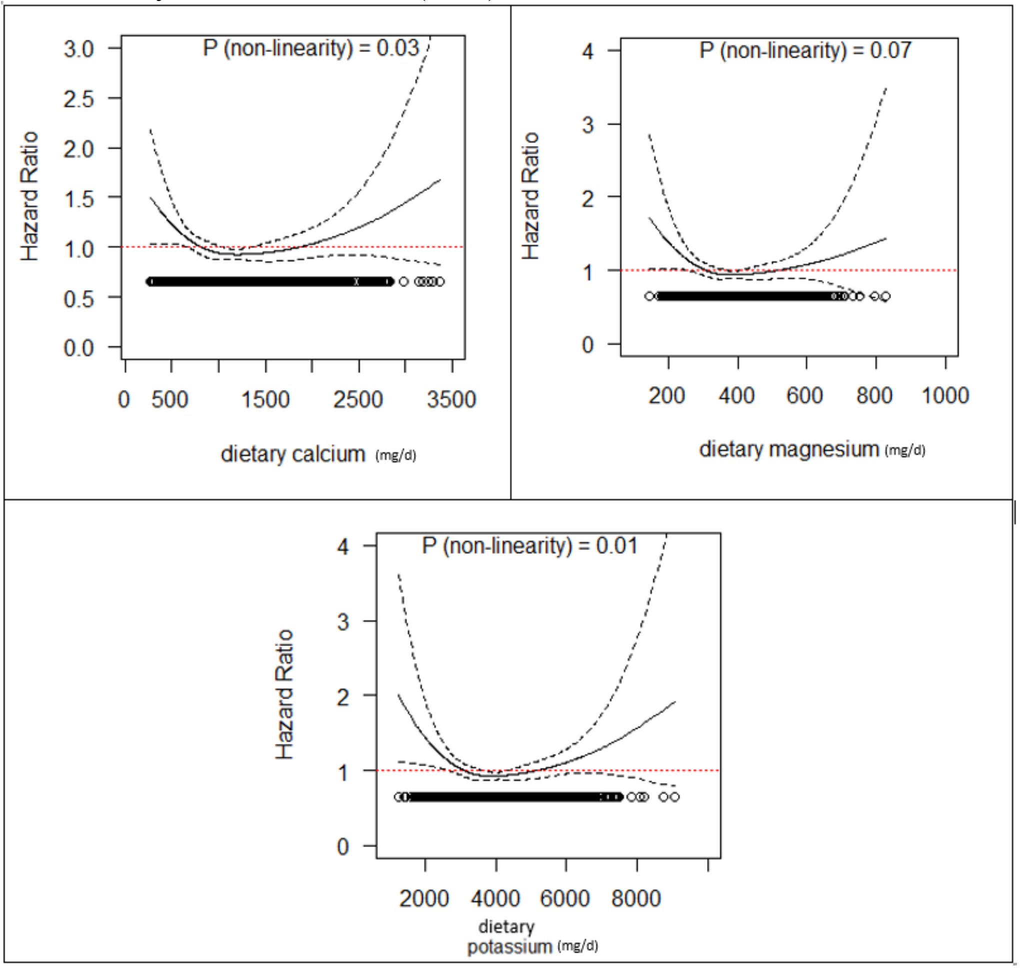

Figure S4. Splines for dietary mineral intake (mg/day) of calcium, magnesium and potassium and stroke

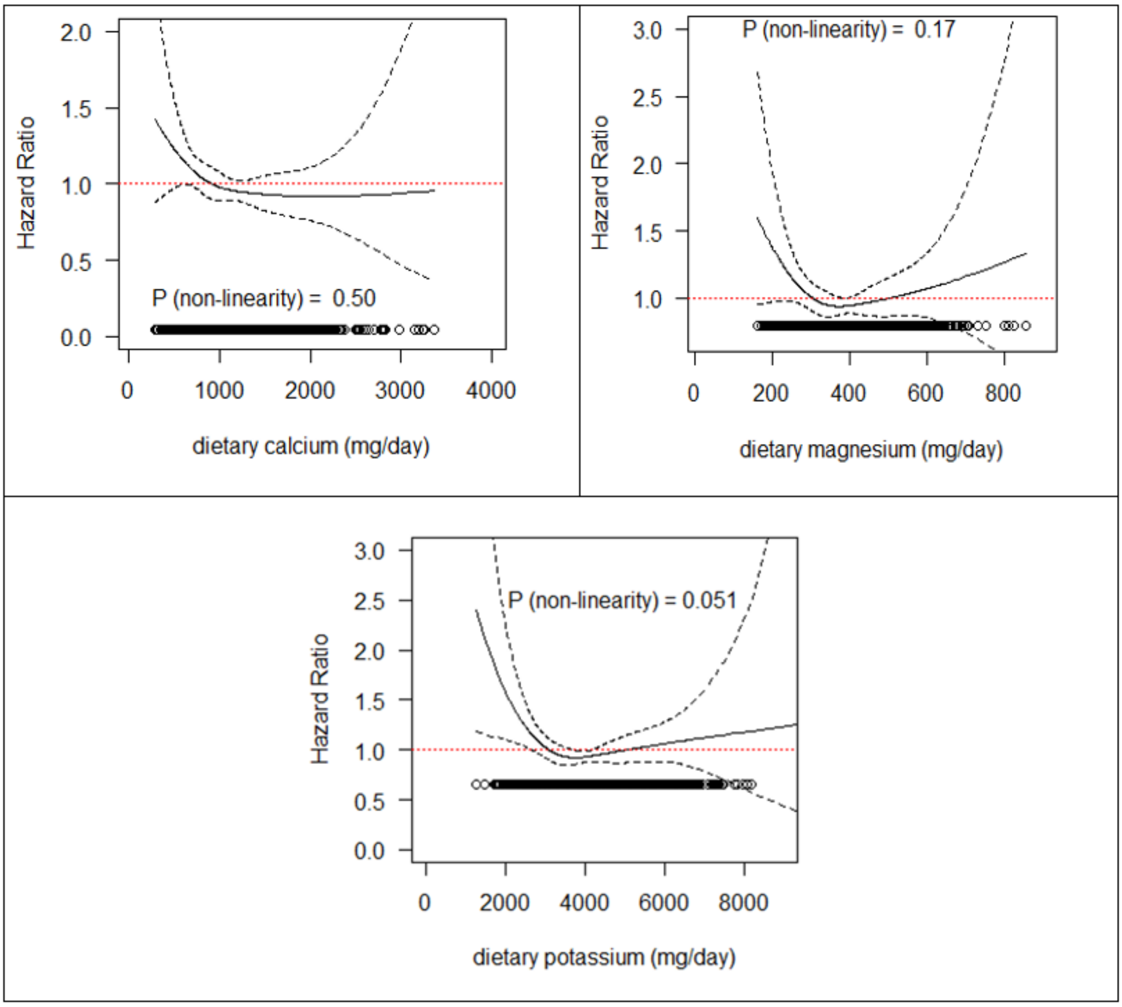

Supplement: Supplementary file 1 [file ijerph-21-00932-s001.zip › ijerph-3068104-supplementary.pdf]
